# Increased Serum Levels of Uric Acid Are Associated with Sudomotor Dysfunction in Subjects with Type 2 Diabetes Mellitus

**DOI:** 10.1155/2011/346051

**Published:** 2011-09-15

**Authors:** N. Papanas, M. Demetriou, N. Katsiki, K. Papatheodorou, D. Papazoglou, T. Gioka, S. Kotsiou, E. Maltezos, D. P. Mikhailidis

**Affiliations:** ^1^Outpatient Clinic of Obesity, Diabetes, and Metabolism, Second Department of Internal Medicine, University Hospital of Alexandroupolis, Democritus University of Thrace, 68100 Alexandroupolis, Greece; ^2^Department of Clinical Biochemistry, Vascular Disease Prevention Clinics, Royal Free Hospital, University College London Medical School, University College London (UCL), London NW3 2QG, UK; ^3^Biochemistry Laboratory, University Hospital of Alexandroupolis, 68100 Alexandroupolis, Greece

## Abstract

The aim of this paper was to assess serum uric acid (SUA) levels in patients with type 2 diabetes mellitus (T2DM) with or without sudomotor dysfunction (evaluated by the Neuropad test). We included 36 T2DM patients with sudomotor dysfunction (group A: mean age 63.1 ± 2.6 years) and 40 age-, gender-, renal function- and T2DM duration-matched patients without sudomotor dysfunction (group B: mean age 62.1 ± 3.1 years). SUA was significantly higher in group A (*P* < 0.001). There was a significant correlation between SUA and Neuropad time to colour change in both groups (group A: *r*_*s*_ = 0.819, *P* < 0.001; group B: *r*_*s*_ = 0.774, *P* < 0.001). There was also a significant positive correlation between SUA and CRP in both groups (group A: *r*_*s*_ = 0.947, *P* < 0.001; group B: *r*_*s*_ = 0.848, *P* < 0.001). In conclusion, SUA levels were higher in T2DM patients with sudomotor dysfunction than those without this complication. The potential role of SUA in sudomotor dysfunction merits further study.

## 1. Introduction

Diabetic autonomic neuropathy (DAN) is a major cause of morbidity and mortality [1, 2]. A rather neglected manifestation of DAN is impaired sweat secretion, also called sudomotor dysfunction [3]. This is due to injury of the postganglionic cholinergic sympathetic nerve fibres which are responsible for the innervation of sweat glands and causes dry skin [3].

Serum uric acid (SUA) is being considered as a risk marker of cardiovascular morbidity [4–9]. Elevated SUA levels are associated with endothelial dysfunction [10], coronary artery disease [4–6], stroke [11, 12], peripheral arterial disease (PAD) [13, 14], as well as cardiovascular mortality [5, 7]. Furthermore, a link between increased SUA levels and nonalcoholic fatty liver disease (NAFLD) has been reported [15]. Specifically, in patients with type 2 diabetes mellitus (T2DM), high SUA levels have been linked with macrovascular disease [16], namely, stroke [17] and PAD [18] while the association between SUA and microvascular disease has received little attention. More recently, however, some data on its relationship with microangiopathy have become available. Indeed, patients with T2DM and peripheral neuropathy have been reported to have higher SUA [19]. There was also an association between SUA levels and clinical severity of neuropathy [19]. 

Despite the reported association between SUA levels and peripheral neuropathy, the potential association with DAN has not been examined. The rationale for this association is that SUA reflects inflammatory activity [9, 10], and patients with DAN may exhibit increased inflammation [20]. In particular, SUA levels have not been examined in diabetic patients with sudomotor dysfunction. Of note, there is evidence linking SUA levels and the amount of sweating, at least in healthy volunteers [21]; however, the effect of sudomotor dysfunction on this relationship is unclear. Thus, we examined the association between SUA and sudomotor dysfunction in patients with T2DM. It is also of interest that both SUA [5, 7] and DAN [1, 2] may predict cardiovascular mortality.

## 2. Patients and Methods

The present study included 36 T2DM patients with sudomotor dysfunction (group A) and 40 T2DM patients without sudomotor dysfunction (group B). These groups were matched for age, gender, renal function (assessed by serum creatinine), and diabetes duration. Patient characteristics are presented in Table 1. Subjects were recruited from the Outpatient Clinic of Obesity, Diabetes and Metabolism, Second Department of Internal Medicine at Democritus University of Thrace, Greece. The study was carried out in accordance with the Helsinki Declaration of Human Rights and patients gave their informed consent.

Exclusion criteria were age <17 or >75 years, chronic renal failure, PAD, stroke, other potential causes of neuropathy (i.e., alcohol abuse, vitamin B12 deficiency, peripheral nerve lesions, or malignancy), acute or chronic infections or inflammation, haematological disease, as well as use of statins, anti-inflammatory agents, antibiotics, or any medication that might influence SUA levels (e.g., diuretics, cyclosporin, allopurinol, oestrogens, or cytotoxic agents) [22–24] during the previous 3 months.

Sudomotor dysfunction was diagnosed by means of the indicator test Neuropad (Trigocare International GmbH, Wiehl, Germany) [25]. This is a relatively new test, which assesses sweat production by means of a chemical reaction between water and anhydrous cobalt dichloride [25, 26]. The test is applied to an area free from callus and hyperkeratosis on the plantar aspect of the foot between the first and second metatarsal head [25]. In case of adequate sweat production, the test changes its colour from blue to pink within 600 sec. If such colour change does not occur within this time frame, sudomotor dysfunction is diagnosed. The severity of the latter can be evaluated by measuring the absolute time to colour change [25, 26]. In the present study, the 600 sec cutoff was used to diagnose sudomotor dysfunction while time to colour change was measured to evaluate its severity [25].

SUA, serum creatinine, C-reactive protein (CRP), triglycerides (TGs), total cholesterol (TC), high-density lipoprotein cholesterol (HDL-C), and low-density lipoprotein cholesterol (LDL-C) were measured by spectrophotometry (Olympus AU 2700 autoanalyzer, Hamburg, Germany). Glomerular filtration rate (GFR) was calculated by the Cockroft-Gault formula. Haemoglobin A_1c_ (HbA_1c_) was assessed by high-performance liquid chromatography (G7 HPLC Glycohemoglobin Analyzer, Tosoh Bioscience, Inc., South San Francisco, CA, USA). Urinary albumin was measured by an immunonephelometric method (Immage 800, Beckman-Coulter, Inc., Brea, CA, USA). 

Smoking was defined as daily consumption of ≥1 cigarette for at least 2 years during the preceding 5 years. In accordance with the seventh report of the Joint National Committee on prevention, detection, evaluation, and treatment of high blood pressure (JNC7), we defined arterial hypertension as a systolic blood pressure ≥140 mmHg and/or diastolic blood pressure ≥90 mmHg on 3 separate occasions [27] or as antihypertensive treatment. Coronary artery disease (CAD) was diagnosed on the basis of history of myocardial infarction and/or electrocardiographic findings [28]. Microalbuminuria was diagnosed when albumin excretion rate was ≥20 *μ*g/min in the absence of uncontrolled hypertension and/or urinary tract infection [29] and recorded as present or absent. Retinopathy was defined as at least 2 microaneurysms and/or retinal haemorrhage and/or other signs of retinal damage [30]. Body mass index (BMI) was calculated by dividing subjects' weight by their squared height (kg/m^2^).

Statistical analysis was performed using the SPSS (Statistical Package for Social Sciences, Chicago, Illinois) version 13.0. Normally distributed quantitative variables were analysed by unpaired *t*-test. Qualitative variables were compared by chi-square (after Yates' correction when appropriate). Correlations between Neuropad time to colour change and/or biochemical variables (i.e., SUA, TG, TC, HDL-C, LDL-C, serum creatinine, GFR, and CRP) were evaluated by Spearman's coefficient (*r*_*s*_). Data were expressed as mean ± standard deviation (SD). A 2-tailed *P* ≤ 0.05 was considered significant.

## 3. Results

There were no differences between the 2 groups in demographic characteristics, glycaemic control (assessed by HbA_1c_), renal function (assessed by serum creatinine and GFR), and frequency of diabetic complications other than neuropathy (Table 1). SUA levels were significantly higher in group A versus B (8.2 ± 1.4 versus 5.8 ± 1.1 mg/dL, *P* < 0.001). The same was observed for CRP (1.0 ± 0.4 versus 0.3 ± 0.2 mg/dL, *P* < 0.001) and TGs (158 ± 24 versus 146 ± 17 mg/dL, *P* = 0.016), but not for TC (177 ± 19 versus 175 ± 18 mg/dL, *P* = 0.83), LDL-C (102 ± 19 versus 102 ± 20 mg/dL, *P* = 0.98), and HDL-C (43 ± 7 versus 44 ± 6 mg/dL, *P* = 0.38).

Overall, there was a significant positive correlation between SUA and Neuropad time to colour change (*r*_*s*_ = 0.874, *P* < 0.001). This correlation was observed both in group A (*r*_*s*_ = 0.819, *P* < 0.001) and in group B (*r*_*s*_ = 0.774, *P* < 0.001). Furthermore, there was a significant positive correlation between SUA and CRP (*r*_*s*_ = 0.935, *P* < 0.001), which was also observed both in group A (*r*_*s*_ = 0.947, *P* < 0.001) and in group B (*r*_*s*_ = 0.848, *P* < 0.001).

A significant correlation between SUA and TGs was observed in group A (*r*_*s*_ = 0.556, *P* < 0.001) but not in group B (*r*_*s*_ = 0.185, *P* = 0.254). Moreover, there was a significant negative correlation between SUA and HDL-C in group A (*r*_*s*_ = −0.400, *P* = 0.016) but not in group B (*r*_*s*_ = 0.291, *P* = 0.130). SUA did not correlate with TC (group A: *r*_*s*_ = 0.285, *P* = 0.920; group B: *r*_*s*_ = 0.156, *P* = 0.335) or LDL-C (group A: *r*_*s*_ = 0.321,  *P* = 0.610; group B: *r*_*s*_ = 0.290, *P* = 0.710). There was no correlation between SUA and HbA_1c_ in either group (group A: *r*_*s*_ = −0.072, *P* = 0.676; group B: *r*_*s*_ = 0.182, *P* = 0.260).

Overall, there was a significant positive correlation between SUA and serum creatinine (*r*_*s*_ = 0.428, *P* = 0.001). This correlation was observed both in group A (*r*_*s*_ = 0.580, *P* = 0.001) and in group B (*r*_*s*_ = 0.370, *P* = 0.029). SUA also correlated negatively with GFR (*r*_*s*_ = −0.510, *P* = 0.001). This correlation was observed both in group A (*r*_*s*_ = −0.601, *P* = 0.002) and in group B (*r*_*s*_ = −0.410, *P* = 0.004).

## 4. Discussion

Our main finding is that SUA levels are increased in the presence of sudomotor dysfunction among patients with T2DM. Of note, the 2 groups were matched for renal function (assessed by serum creatinine and GFR), as well as age, diabetes duration, and HbA_1c_. These novel findings extend our previous observation of elevated SUA in patients with peripheral diabetic neuropathy [19]. Hence, it appears that the association between SUA and diabetes-related neuropathy exists both for peripheral neuropathy and for sudomotor dysfunction. Importantly, there was a significant positive correlation between SUA and Neuropad time to colour change, a marker of the severity of sudomotor dysfunction [26]. This correlation was observed in both groups, as well as among the entire patient population, suggesting a continuum of increasing SUA levels with progression of sudomotor dysfunction.

Moreover, patients with sudomotor dysfunction exhibited significantly higher CRP levels than those without sudomotor dysfunction. There was a significant positive correlation between CRP and SUA levels in both groups and in the entire patient population. An association between neuropathy and CRP has been reported by us [19] and others [21, 31]. The role of inflammation in diabetic neuropathy has also become apparent by the observation of elevated interleukin-6 levels in the presence of this complication [31, 32]. Our results provide further support for the role of inflammation in neuropathy, as well as the first suggestion that inflammation is important in the context of sudomotor dysfunction. As SUA has been shown to exert antioxidant properties [33, 34], it is possible that inflammatory activity may induce an elevation of SUA levels. 

Another finding was the association of sudomotor dysfunction with increased TGs and reduced HDL-C. We have previously shown that peripheral neuropathy is associated with increased TGs and TC, but not HDL-C, in T2DM [19]. Despite some minor differences in the lipid fractions involved, these findings highlight the importance of dyslipidaemia in diabetic neuropathy. Our results are in agreement with experimental evidence that elevated TG and oxidised LDL are neurotoxic via oxidative stress, independently of hyperglycaemia [35]. Others have also implicated hyperlipidaemia in diabetic neuropathy and identified elevated serum lipids as a potential additional therapeutic target in the management of this microvascular complication [36–38]. These observations may explain why SUA correlated with dyslipidaemia but not with HbA_1c_.

The limitations of the study include that it was not prospective, and thus the potential role of SUA in the development of sudomotor dysfunction could not be examined. Secondly, we assessed a relatively small number of patients and they were recruited in a specialist unit. Therefore, caution is needed before applying our results to the general T2DM population. Furthermore, Neuropad has not yet been established as the gold standard for the assessment of sudomotor dysfunction. However, the more sophisticated modalities, such as the quantitative sudomotor axon reflex test, are only available in a handful of specialised centres across the world and are far too cumbersome to use in clinical practice [3]. Neuropad is the only widely available test for broad assessment of sudomotor dysfunction and dry foot [26]. Of note, the literature on the application of Neuropad is ever increasing [38–40], lending further support to its use for the evaluation of this manifestation of neuropathy. Finally, no information on nutritional habits was available, and so the potential contribution of dietary factors could not be accurately evaluated. However, all patients received dietary advice as part of their routine care. 

Our results may have the following practical implications. T2DM patients with sudomotor dysfunction exhibit significantly higher SUA than those without. SUA levels are elevated in association with the severity of sudomotor dysfunction and they are associated with increased CRP and dyslipidaemia. Based on these findings, SUA may be considered a potentially useful additional marker for sudomotor dysfunction. This role should be discussed in the context of the emerging evidence for the contribution of inflammation [19, 21, 31, 32], hyperlipidaemia [19, 35–37, 41], and other factors to the pathogenesis of diabetes-related neuropathy, highlighting the necessity for multifactorial intervention [42, 43]. Nonetheless, 3 important issues remain to be clarified. First, the putative contribution of SUA to the development of sudomotor dysfunction needs to be examined in long-term prospective studies. Secondly, we need to investigate whether SUA is elevated in patients with other manifestations of DAN, notably reduced heart rate variability or gastroparesis. Finally, it remains to be established whether pharmacological reduction of SUA might contribute to the management and/or prevention of sudomotor dysfunction, as may be possible for cardiovascular disease [9, 11, 33] and metabolic syndrome [44]. 

In conclusion, SUA levels are higher in T2DM patients with sudomotor dysfunction compared with those without this complication. A significant positive correlation was observed between SUA and the severity of sudomotor dysfunction. Furthermore, SUA levels are associated with CRP. In subjects with sudomotor dysfunction, SUA also correlates with an adverse lipid profile. The present findings suggest that prospective studies should further examine the contribution of SUA to the development of sudomotor dysfunction.

## Figures and Tables

**Table 1:** Characteristics of patients with (group A) versus without (group B) sudomotor dysfunction.

Characteristic	Group A (*n* = 36)	Group B (*n* = 40)	*P *
Men (*n*, %)	17 (47.2%)	19 (47.5%)	0.981
Age (years, mean ± SD)	63.1 ± 2.6	62.1 ± 3.1	0.251
Serum creatinine (mg/dL, mean ± SD)	0.9 ± 0.1	0.8 ± 0.1	0.322
eGFR (mL/min, mean ± SD)	85 ± 5	86 ± 5	0.508
Diabetes duration (years, mean ± SD)	9.4 ± 2.1	8.9 ± 1.9	0.246
HbA_1c_ (%, mean ± SD)	7.7 ± 0.4	7.6 ± 0.5	0.750
BMI (kg/m^2^, mean ± SD)	31.5 ± 1.8	32.1 ± 1.8	0.210
Smoking (*n*, %)	13 (36.1%)	13 (32.5%)	0.929
Hypertension (*n*, %)	27 (75%)	31 (77.5%)	0.999
CAD (*n*, %)	12 (33.3%)	13 (32.5%)	0.999
Retinopathy (*n*, %)	18 (50%)	24 (60%)	0.519
Microalbuminuria (*n*, %)	16 (44.4%)	12 (30%)	0.287
